# Fermented fish oil suppresses T helper 1/2 cell response in a mouse model of atopic dermatitis via generation of CD4^+^CD25^+^Foxp3^+^ T cells

**DOI:** 10.1186/1471-2172-13-44

**Published:** 2012-08-09

**Authors:** Sang-Chul Han, Gyeoung-Jin Kang, Yeong-Jong Ko, Hee-Kyoung Kang, Sang-Wook Moon, Yong-Seok Ann, Eun-Sook Yoo

**Affiliations:** 1 Department of Pharmacology, School of Medicine, Jeju National University, 66 Jejudaehakno, Jeju, 690-756, South Korea; 2 Fermentec Inc, 207, Jeju Bio industrial Center, 66 Jejudaehakno, Jeju, 690-756, South Korea; 3 Choung Ryong Fisheries Co. LTD, Seogwipo-city, Jeju, 697-943, South Korea

## Abstract

**Background:**

Allergic skin inflammation such as atopic dermatitis (AD), which is characterized by pruritus and inflammation, is regulated partly through the activity of regulatory T cells (Tregs). Tregs play key roles in the immune response by preventing or suppressing the differentiation, proliferation and function of various immune cells, including CD4^+^ T cells. Recent studies report that fermentation has a tremendous capacity to transform chemical structures or create new substances, and the omega-3 polyunsaturated fatty acids (n-3 PUFAs) in fish oil can reduce inflammation in allergic patients. The beneficial effects of natural fish oil (NFO) have been described in many diseases, but the mechanism by which fermented fish oil (FFO) modulates the immune system and the allergic response is poorly understood. In this study, we produced FFO and tested its ability to suppress the allergic inflammatory response and to activate CD4^+^CD25^+^Foxp3^+^ Tregs.

**Results:**

The ability of FFO and NFO to modulate the immune system was investigated using a mouse model of AD. Administration of FFO or NFO in the drinking water alleviated the allergic inflammation in the skin, and FFO was more effective than NFO. FFO treatment did increase the expression of the immune-suppressive cytokines TGF-β and IL-10. In addition, ingestion of FFO increased Foxp3 expression and the number of CD4^+^CD25^+^Foxp3^+^ Tregs compared with NFO.

**Conclusions:**

These results suggest that the anti-allergic effect of FFO is associated with enrichment of CD4^+^CD25^+^ Foxp3^+^ T cells at the inflamed sites and that FFO may be effective in treating the allergic symptoms of AD.

## Background

Atopic dermatitis (AD), a chronic inflammatory skin disease associated with cutaneous hyper-reactivity, affects approximately 3% of infants, 10 ~ 20% of children and 1 ~ 3% of adults worldwide [1]. This systemic disorder is caused by skin barrier dysfunction, severe skin dehydration, and mutations in the filaggrin gene, which has an essential role in modulating epidermal homeostasis. The skin lesions of AD are generally characterized by infiltration with various inflammatory cells such as mast cells, basophils, eosinophils and T cells [2-5].

Activated mast cells release a variety of inflammatory mediators following cross-linking of immunoglobulin E (IgE)-receptor complexes at the high-affinity IgE receptor I (FcεRI). Of these mediators, histamine is generally considered as a marker of mast cell degranulation in immediate allergic reaction and is a violent inducer of itching. Histamine is a characteristic major mediator in mast cell storage granules and directly triggers type I allergic responses [6-10].

Thymic stromal lymphopoietin (TSLP) is produced mainly by epidermal keratinocytes, as well as dermal fibroblasts and mast cells in the skin lesions of acute and chronic AD [11]. It strongly promotes dendritic cell (DC) maturation in the epidermis or can initiate and regulate the allergic inflammation reaction [12,13]. Moreover, TSLP can stimulate naive T cell to express pro-inflammatory cytokines (IL-4, -5 and −13) [14].

Regulatory T cells (Tregs) play a key role in various immune responses, including type 2 helper T cells (Th2)-mediated diseases, such as AD. Tregs are found in lymph nodes, skin lesions, spleen or peripheral blood and maintain peripheral immune homeostasis and tolerance to allergens. They also prevent or suppress differentiation, proliferation and function of various immune cells, including CD4^+^ and CD8^+^ T cells, through a process dependent on cell-cell contact, IL-10, or transforming growth factor (TGF-β) [15-19]. Exhaustion of CD25^+^ cells can aggravate Th2 cell-mediated inflammation induced by various antigens [20]. Tregs express high CD25 levels at the cell surface and CD25 has been identified as a marker of Tregs. However, CD25^hi^ expression is regarded as an activation marker rather than a normal marker of Tregs because CD25 expression on Tregs increases after sensitization with antigens [21]. The expression of forkhead box protein 3 (Foxp3), which is a key regulator of Treg development, has been identified as a specific Treg marker. Foxp3^+^ T cells can inhibit the activation or proliferation of various immune cells, including Th1, Th2 and Th17 cells [22].

Several previous studies have examined the anti-allergic effects of fish oil. Fish oil intake can reduce sensitization to allergens, alleviate the severity of AD, eczema, and asthma, and down-regulate the expression of IL-1, -4, and −13 and IFN-γ in serum [23,24]. Fish oil contains various Omega-3 polyunsaturated fatty acid (n-3 PUFAs), such as eicosapentaenoic acid (EPA) and docosahexaenoic acid (DHA) [25]. EPA inhibits the production of pro-inflammatory cytokines (IL-2, IL-12 and IFN-γ), up-regulates the expression of anti-inflammatory cytokines (IL-10), and increases the number of Foxp3^+^ Tregs [26]. DHA diminishes the proliferation of effector T cells by increasing mRNA expression of Foxp3, TGF-β and IL-10 from Tregs [27].

Recent studies have investigated the anti-allergic effects of fermented food products such as yogurt, cheese, and soybean paste on a variety of allergic reactions, diabetes and some cancers [28-30]. Fermentation can transmute the chemical structure of some constituents to create new substances. The beneficial effects of natural fish oil (NFO) have been described in many diseases, but the mechanism by which fermented fish oil (FFO) modulates the immune system and the allergic response is poorly understood. In this study, we produced FFO and found that ingestion of this fish oil resulted in suppression of various allergic reactions and factors and increased the differentiation of CD4^+^CD25^+^Foxp3^+^ T cells from CD4^+^ T cells.

## Results

### FFO suppresses causative factors in pruritus

**Figure 1:**
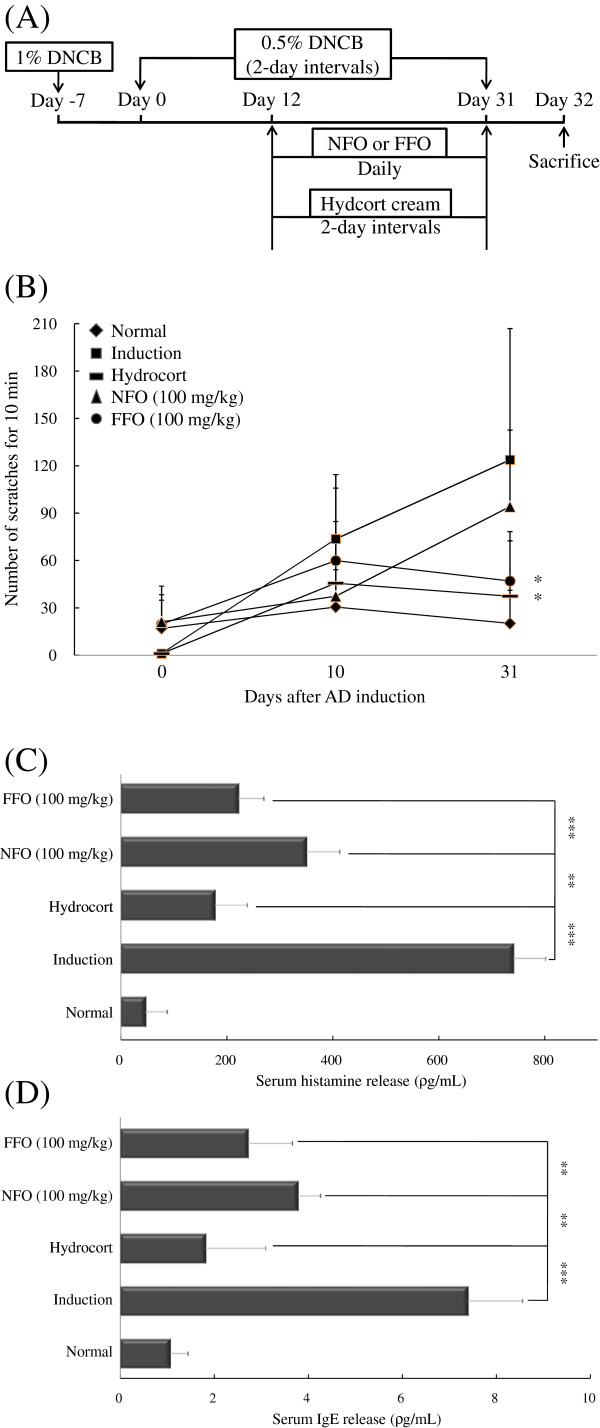
**FFO suppresses causative factors in pruritus.** (**A**) Mice were painted with 1% DNCB or vehicle on their abdomen as the first sensitization (day-7). On day 0, the mice were sensitized with 0.5% DNCB on their ears and were then sensitized with 0.5% DNCB every other day for up to 31 days. From day 12 until the completion of the experiment, mice were given FFO or NFO (100 mg/kg) in their drinking water, daily, and were painted with hydrocort on their ears every other day. The mice were sacrificed on day 32. (**B**) Mice were given 100 mg/kg FFO (•) or NFO (▴) daily and painted with hydrocort (−) every other day. The spontaneous scratching behaviors of the mice were recorded for 10 min with a digital camera on days 0, 10 and 31. (**C** and **D**) After sacrifice, the histamine and IgE in mouse serum was measured by ELISA. Data are representative of 8 mice per group. Values are mean ± S.D (n = 8 mice per group). **, *** compared to mice stimulated with DNCB alone (induction group). **P < 0.005; *** *P* < 0.001.

To induce experimental AD, mice received an initial sensitization with 1% dinitrochlorobenzene (DNCB) on the abdomen, followed by sensitization with 0.5% DNCB on their ears every other day for up to 31 days. Starting on day 12, the mice were given 100 mg/kg of FFO or NFO orally every day. On day 32, all mice were sacrificed (Figure 1A). Itching is a critical factor in the maintenance of AD symptoms and affects skin edema. Therefore, we tested whether FFO or NFO has a therapeutic effect on pruritus. Mice with experimental AD were given FFO or NFO (100 mg/kg) every day, and the mice were painted with hydrocort cream every other day. We measured the number of scratches in a 10 min period on days 0, 10 and 31. In the induction group, the number of scratches increased in a time-dependent manner. Also, on day 31, FFO and hydrocort cream treatment significantly decreased (*P* < 0.05) scratching behavior (Figure 1B).

IgE is an important therapeutic target for allergy and AD as it is the major mechanism for activating mast cells to release histamine [31]. Therefore, we measured serum IgE and histamine levels in mice with dermatitis by Enzyme-linked immunosorbent assay (ELISA). The FFO or NFO-treated group showed significantly decreased IgE (both *P* < 0.005) and histamine (each *P* < 0.001 or *P* < 0.005) levels compared with the induction group. In addition, FFO treatment had a stronger inhibitory effect than NFO on allergic symptoms (Figure 1C and D).

### Histological features of ear tissue and TSLP hyperproduction in DNCB-challenged mice

**Figure 2:**
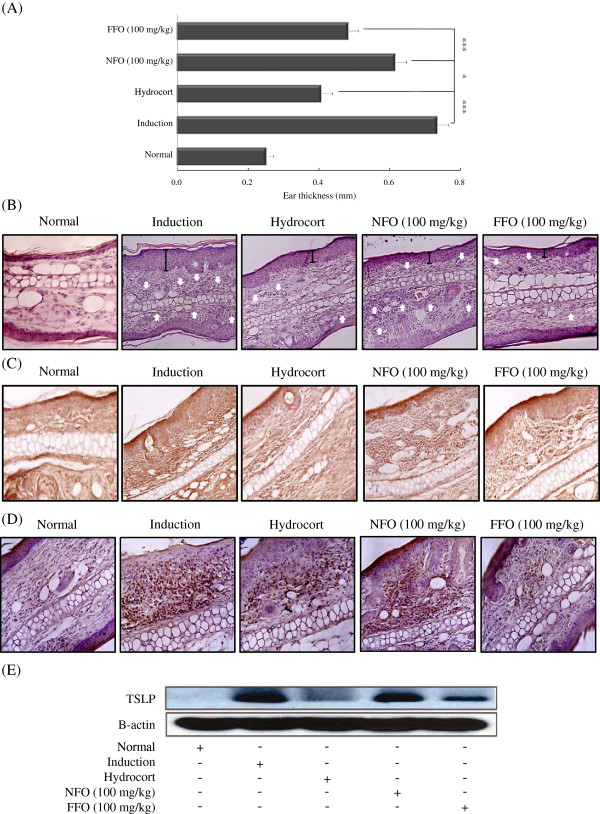
**Histological features of ear tissue and TSLP hyperproduction in DNCB-challenged mice.** (**A**) BALB/c mice were sensitized with DNCB at 2-day intervals for up to 31 days, given 100 mg/kg FFO or NFO daily, and painted with hydrocort every other day, as described in Figure legend 1. Ear thickness was measured on day 31. (**B**) Paraffin sections of ear tissues were stained with hematoxylin and eosin (H&E, ×200), and (**C**) processed for CD3^+^ cells and (**D**) TSLP immunohistochemistry (IHC, ×200). (**E**) TSLP was measured in ear tissues by western blot. Data are representative of 8 mice per group. Values are mean ± S.D (n = 8 mice per group). *P < 0.05 and ***P < 0.001 compared to mice stimulated with DNCB alone (induction group).

The skin lesions of AD are characterized by infiltration of various inflammatory cells such as lymphocytes, granulocytes, and T cells [32]. Therefore, we tested whether administration of FFO or NFO alleviates inflammatory cell infiltration in the ears of mice with experimental AD. Cutaneous edema as a measure of AD progression was also measured. Administration of FFO (*P* < 0.001) or NFO (*P* < 0.05) reduced ear thickness; the FFO-treated group was not appreciably different from the hydrocort cream treatment group (Figure 2A). The effect of FFO and NFO treatment on infiltration of inflammatory cells into the ear tissue was monitored by hematoxylin and eosin (H&E) staining. The FFO and NFO-treated group showed significantly decreased epidermal thickness and inflammatory cell infiltration such as CD3^+^ cells compared with the induction group (Figure 2B and C). 

TSLP produced by epidermal keratinocytes, dermal fibroblasts and mast cells can activate the maturation of dendritic cells and regulate allergic inflammation reactions. Therefore, we measured the expression of TSLP in AD mice models using IHC and western blot. The increased TSLP expression was significantly inhibited in the FFO group (Figure 2D and E). Although both fish oils inhibited various feature of AD mice models, FFO administration showed stronger inhibitory effects than NFO on cutaneous edema, CD3^+^ T cell infiltration and TSLP hyperproduction.

### FFO administration regulates the expression of AD-associated cytokines and the Foxp3 transcription factor

**Figure 3:**
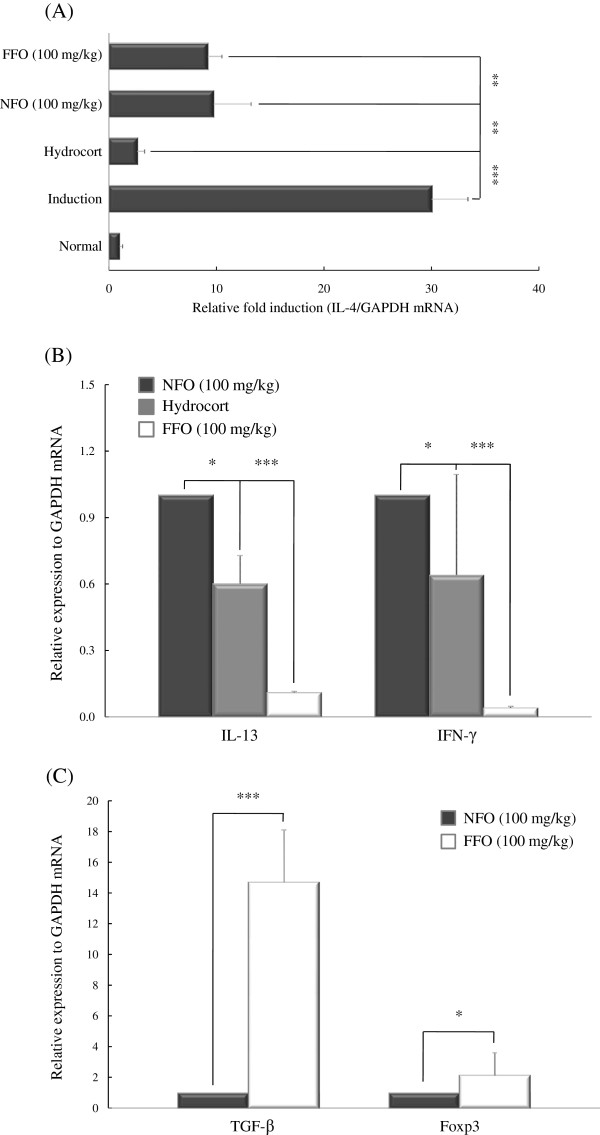
**FFO administration regulates the expression of AD-associated cytokines and the Foxp3 transcription factor.** Mice with experimental AD were given 100 mg/kg FFO or NFO daily and painted with hydrocort every other day, as described in Figure legend 1. After sacrifice, ear tissues were isolated and used to measure the mRNA expression of inflammatory cytokines including (**A**) IL-4, (**B**) IL-13 and INF-γ, and (**C**) TGF-β and Foxp3 by real-time PCR. Data are representative of 8 mice per group. Values are mean ± S.D (n = 8 mice per group). *P < 0.05; ** *P* < 0.005; and *** *P* < 0.001 compared to mice in induction group (**A**) or NFO-treated group (**B** and **C**).

T cells produce a variety of cytokines. Th1 cells are characterized by the production of inflammatory cytokines such as IFN-γ and IL-2 and Th2 cells are characterized by the production of various cytokines including IL-4, -5 and −13. Among these cytokines, IL-4 plays a crucial role in allergic responses and in the differentiation from naive T cell to Th2 cell. IL-4 also induces isotype switching of B cells to produce IgE [33]. We measured whether FFO affects the expression of pro-inflammatory cytokines in DNCB-induced dermatitis, which is characterized as a Th2 inflammatory disorder. The expression of AD-associated inflammatory factors such as Th2 (IL-4, -13) and Th1 (IFN-γ) cytokines was measured in the ear tissues of DNCB-stimulated mice by real-time PCR. In the induction group, the IL-4 level was extremely elevated. Both FFO and NFO decreased the expression of IL-4 compared with the induction group (both *P* < 0.005); the effect of FFO was not appreciably different from that of NFO (Figure 3A). Ingestion of FFO reduced IL-13 (*P* < 0.001) and IFN-γ (*P* < 0.001) mRNA levels compared with the NFO-treated group (Figure 3B). We also measured the effects of FFO and NFO on the expression of TGF-β and Foxp3, both of which induce the proliferation and differentiation of Tregs. FFO treatment increased the expression of TGF-β and Foxp3 compared with NFO group (*P* < 0.001 and *P* < 0.05, respectively) (Figure 3C). 

### FFO attenuates the function of mature Th1/2 cells in experimental immune disorders

**Figure 4:**
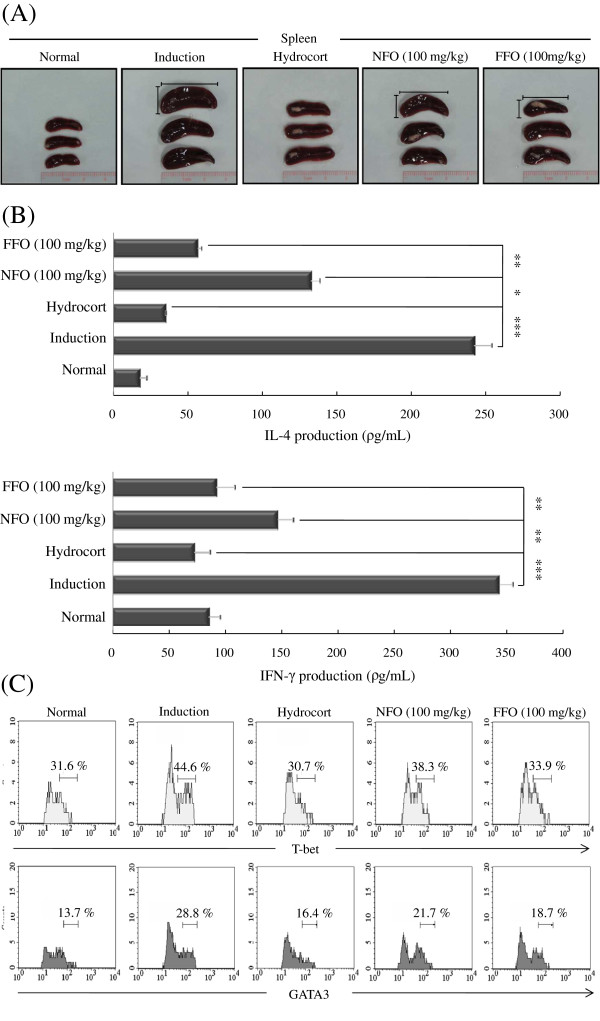
**FFO attenuates mature Th1/2 cell functions in experimental atopic dermatitis.** (**A**) Mice were stimulated with DNCB for up to 31 days at 2-day intervals, given 100 mg/kg FFO or NFO every day, and were painted with hydrocort every other day, as described in Figure legend 1. After sacrifice, the spleen was isolated and photographed to record the morphologic alteration. (**B**) Splenocytes were isolated from mice in each experimental group and 1.0 × 10^6^ cells/mL were stimulated with anti-CD3 (1 μg/mL) and anti-CD28 (0.5 μg/mL) for 3 days. Mouse IL-4 and IFN-γ were measured in the culture supernatant by ELISA. (**C**) Cells were permeabilized with T-bet fixation/permeabilization buffer and stained with anti-T-bet-Alexa Fluor® 488 and anti-GATA3-PE and analyzed by FACS. Data are representative of 8 mice per group. The measurements were made in triplicate and are shown as mean ± S.D (n = 8 mice per group). (**B**) *P < 0.05; **P < 0.005; and *** *P* < 0.001 compared to mice sensitized with DNCB alone (induction group).

The spleen plays important roles in the active immune system through the cell-mediated pathway, especially through the activity of mature T and B cells [34]. Therefore, we observed the morphologic features of the spleen. Mice in induction group showed a highly enlarged spleen. FFO and NFO treatment resulted in a smaller spleen size with reduced the thickness and length compared to the induction group, and the FFO group had a smaller spleen than the NFO group (Figure 4A). 

We measured whether FFO or NFO suppresses mature Th1 and Th2 cell functions in the experimental AD models. Splenocytes from each group were stimulated with anti-CD3 (1 μg/mL) and anti-CD28 (0.5 μg/mL) for 3 days. The relative levels of IFN-γ and IL-4 cytokines were measured by ELISA and the expression of the Th1 T-box-expressed-in-T-cell (T-bet) and Th2 GATA-binding protein 3 (GATA3) transcription factors was measured by FACS. Spleen cells from mice treated with FFO and NFO produced significantly less IL-4 and IFN-γ compared with the induction group following stimulation with anti-CD3/CD28 (Figure 4B). In addition, ingestion of FFO or NFO decreased T-bet expression from 44.6% of splenocytes (induction group) to 33.9% (FFO) or 38.3% (NFO) and decreased GATA3 expression from 28.8% of splenocytes (induction group) to 18.7% (FFO) or 21.7% (NFO). The FFO group exhibited stronger anti-inflammatory effects on effector T cell inflammatory factors than the NFO group (Figure 4C).

### FFO administration increases the differentiation of CD4^+^CD25^+^Foxp3^+^ Treg

**Figure 5:**
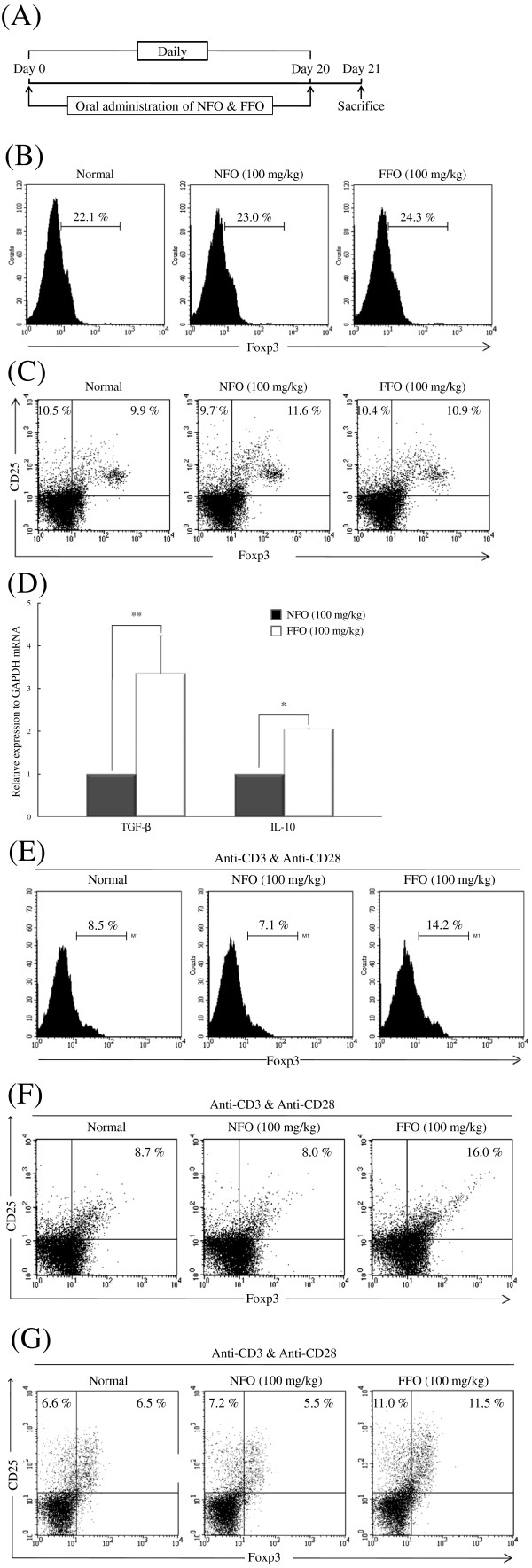
**FFO administration promotes the differentiation of CD4**^**+**^**CD25**^**+**^**Foxp3**^**+**^**Treg.** (**A**) Naïve mice were given 100 mg/kg FFO or NFO in their drinking water, daily, for 20 days. The mice were sacrificed on day 32. (**B**) Splenocytes were isolated from the spleens of each group and 1.0 × 10^6^ cells/mL were permeabilized with Foxp3 fixation/permeabilization buffer and stained with anti-Foxp3-FITC and (**C**) anti-CD25-PE and anti-Foxp3-FITC. The Foxp3 and CD25^+^Foxp3^+^ Treg population was analyzed by FACS. Data are from five mice per group. (**D**) The expression of TGF-β and IL-10 mRNA in the spleen was measured by real-time PCR. (**E**) Splenocytes (1.0 × 10^6^ cells/mL) were stimulated with anti-CD3 (1 μg/mL) and anti-CD28 (0.5 μg/mL). After 3 days, the cells were permeabilized with Foxp3 fixation/permeabilization buffer and stained with anti-Foxp3-FITC. (**F**) The cells from panel (**E**) were stained with anti-CD4-FITC and anti-CD25-PE. (**G**) CD4^+^ T cells were isolated from splenocytes using CD4^+^ T cell isolation beads. The CD4^+^ T cells (1.0 × 10^6^ cells/mL) were stimulated with anti-CD3 (1 μg/mL) and anti-CD28 (0.5 μg/mL) for 3 days, after which they were permeabilized with Foxp3 fixation/permeabilization buffer and stained with anti-Foxp3-FITC and anti-CD25-PE. All data (panels A-G except for D) are from FACS analysis. Data are representative of five mice per group. In panel (**D**), the values are mean ± S.D (n = 5 mice per group). *P < 0.05; and **P < 0.005 compared to mice treated with NFO.

The administration of FFO significantly reduced the progression of experimental AD model by inhibiting various inflammatory processes. Tregs play a key role in various immune responses in preventing or suppressing differentiation, proliferation and function of various immune cells including CD4^+^ T cells. Therefore, we analyzed whether administration of FFO or NFO affects the production of factors related to the generation of Tregs. FFO or NFO (100 mg/kg) was fed to normal BALB/c mice for 20 days and all mice were sacrificed on day 21 (Figure 5A). Splenocytes from each group were analyzed without any stimulation. The administration of FFO did not affect Foxp3 expression levels and the number of CD25^+^Foxp3^+^ Tregs (Figure 5B and C) compared with the normal or NFO group. However, the FFO group did have significantly increased expression of Treg-associated anti-inflammatory factors TGF-β and IL-10 compared with the NFO group (*P* < 0.005 or *P* < 0.01, respectively) (Figure 5D).

The above results suggest that the activity of Tregs was somehow enhanced or altered by FFO. Therefore, we analyzed whether administration of FFO or NFO increases the expression of factors related to Treg differentiation during T cell activation. CD4^+^ T cells were isolated from splenocytes using CD4^+^ T cell isolation beads and were stimulated with anti-CD3 and anti-CD28 for 3 days. FFO administration increased Foxp3 expression (14.2%) and the number of CD4^+^CD25^+^ Tregs (16.0%) to levels more than double that in the NFO group (7.1% and 8.0%, respectively) (Figure 5E and F). In addition, to confirm that the cells were genuine Tregs, we analyzed whether administration of FFO increases the number of CD4^+^CD25^+^Foxp3^+^ Tregs following anti-CD3/anti-CD28 stimulation. FFO treatment increased the CD4^+^CD25^+^Foxp3^+^ Treg population (11.5%) to more than twice that of the NFO group (5.5%), (Figure 5G).

## Discussion

In this study, we fermented natural fish oil and compared the differences between FFO and NFO on the modulation of the immune system. Administration of FFO potently induced the generation of CD4^+^CD25^+^ Foxp3^+^ Treg in the spleen compared with NFO treatment. Increased TGF-β and Foxp3 at sites of inflammation was associated with elevated CD4^+^CD25^+^Foxp3^+^ Treg populations, which can down-regulate the progression of experimental immune disorders. The ingestion of FFO generated regulatory T cell populations.

AD mainly appears together with a variety of diseases such as eczema, asthma and allergic conjunctivitis. The symptoms of AD include pruritus, peripheral eosinophilia, epidermal hyperplasia and tissue remodeling, such as spongiosis.

In the present study, we used the DNCB-induced experimental AD mouse model to investigate the anti-inflammatory effect of FFO or NFO. IgE is an important target in the treatment of allergy, and signaling through FcεRI can induce histamine release from mast cells, which leads to potent induction of pruritus. Scratching behavior is a typical consequence of pruritus and aggravates the symptoms of dermatological diseases such as AD. Therefore, we tested whether FFO or NFO can reduce scratching behaviors, serum histamine, IgE hyperproduction and cutaneouse edema. The administration of FFO or NFO decreased scratching behavior, IgE, histamine levels and edema compared with the induction group (Figures 1 and 2A). We predicted that the decreased IgE and histamine levels after FFO or NFO treatment would lead to alleviation of pruritus and cutaneous edema. H&E staining and IHC of the ear tissue confirmed that ingestion of FFO or NFO alleviated inflammatory cell infiltration (CD3^+^ cells) and expression of TSLP compared with the induction group (Figure 2B-E). Furthermore, the FFO treatment had stronger inhibitory effects than NFO treatment on most of these signs of allergic disease.

AD is generally characterized by increased numbers of activated CD4^+^ or CD8^+^ T cells, and enormous numbers of infiltrating CD4^+^ T cells in the dermis. In the majority of patients, AD is primarily associated with a dysfunction of the body’s immune. In particular, IL-4 plays a crucial role in allergic responses and the differentiation of Th2 cells from naïve T cells [35]. Thus, we assessed whether FFO or NFO has anti-inflammatory effects on AD model, which is a Th2-mediated immune disorder. The induction group had extremely elevated IL-4 levels, which were decreased in the FFO and NFO groups. In addition, ingestion of FFO markedly reduced IL-13 and IFN-γ levels and increased the expression of specific Treg cells markers (TGF-β and Foxp3) compared with NFO group in DNCB-stimulated atopic dermatitis mice model (Figure 3). Based on these results, we concluded that FFO and NFO have potent anti-inflammatory properties, although the activity of FFO was greater than that of NFO. Increased expression of TGF-β and Foxp3 by FFO treatment at the sites of inflammation could reflect the increased number of Foxp3^+^ Tregs.

The spleen plays an important role in regulating the immune system and contains a range of immune cells. Also, enlared spleen means a splenomegaly by abnormality of immune system function. We observed the morphologic features of the spleen in AD model. The induction group had markedly enlarged spleens; spleen size was reduced in the FFO and NFO-treated groups. Mice in the FFO group had smaller spleens than mice in the NFO group (Figure 4A). So, FFO reduced enlarged spleen by alleviating abnormality of immune system function. Th2 cells produce a variety of cytokines, such as IL-4, -5 and −13, and preferentially express GATA3, which is important for Th2 differentiation. Th1 cells produce IFN-γ and the transcription factor (T-bet) is critical for the differentiation of Th1 cells [36]. We measured whether FFO and NFO ingestion regulate the expression of Th1/Th2 associated factors in experimental AD model. FFO and NFO decreased IFN-γ and IL-4 levels and the expression of T-bet and GATA3 (Figure 4B-D).

How does FFO suppress a variety of immune response in DNCB-stimulated experimental AD? Regulatory T cells (Tregs) play a key role in various immune responses, including Th2 cell-mediated diseases such as AD, and prevent or suppress differentiation, proliferation, and function of various immune cells including CD4^+^ T cells in a TGF-β/IL-10/cell contact-dependent manner. Since all Tregs express CD25 at the cell surface and produce the Foxp3 transcription factor, these proteins have been identified as markers of Tregs [37]. We analyzed whether the administration of FFO affects the expression of factors related to Tregs differentiation. Although FFO treatment did not increase the Foxp3 level and there was no difference in the number of CD25^+^Foxp3^+^ Tregs compared to the NFO treatment group, the FFO group did show increased expression of the immune-suppressive cytokines TGF-β and IL-10 (Figure 5B-D). Interestingly, FFO administration more than doubled the expression of TGF-β and Foxp3 at the site of inflammation (Figure 3C). Therefore, we analyzed whether administration of FFO and NFO increases the expression of factors related to Tregs differentiation following stimulation. FFO increased Foxp3 level (14.2%) and the CD4^+^CD25^+^ Tregs population (16.0%) more than two-fold compared with NFO in CD4^+^T cells stimulated with anti-CD3 and anti-CD28 (Figure 5E and F). In addition, we analyzed whether administration of FFO or NFO increases the number of CD4^+^CD25^+^Foxp3^+^ Tregs following stimulation. FFO treatment increased the CD4^+^CD25^+^Foxp3^+^ Tregs population (11.5%) more than two-fold compared with NFO treatment (5.5%) (Figure 5G). As a result, ingestion of FFO significantly increased Foxp3 expression and induced the differentiation of CD4^+^CD25^+^Foxp3^+^ Tregs from CD4^+^ T cells. These results indicate that FFO is more effective than NFO in disease suppression and inducing CD4^+^CD25^+^Foxp3^+^ Tregs.

Why does FFO show more potent anti-allergic effects than NFO? Fish oil contains a variety of substances, such as stearic acid, eicosatrienoic acid, linolenic acid, EPA and DHA [38]. Especially, EPA and DHA are mainly effective for skin inflammation. Several previous studies have suggested that EPA and DHA diet alleviate ear oedema and inhibit the production of pro-inflammatory factors (IκB, MAPKs, TNF-α, IL-6 and COX-2) in experimental skin inflammation models [39-41]. FFO contains more than double the concentration of EPA and DHA (Table 1). In addition, fermentation would transmute the chemical structure of some constituents of fish oil to create new substances, which have immune-modulatory activities by regulating the development of CD4^+^CD25^+^Foxp3^+^ Tregs. 

**Table 1:** Fatty acid composition of natural fish oil and fermented fish oil

**Constituent**	**Natural fish oil (%)**	**Fermented fish oil (%)**
**Myristic acid C14:0**	**3.430**	**2.954**
**Stearic acid C18:0**	**3.417**	**2.992**
**Eicosatrienoc acid C20:3**	**0.523**	**0.497**
**Linolenic acid C18:3n3**	**0.638**	**0.847**
**Eicosatrienoc acid (EPA) C20:5n3**	**2.657**	**4.350**
**Docosahexaenoic acid (DHA) C22:6n3**	**8.366**	**13.614**

In summary, FFO had stronger inhibitory effects on various experimental AD symptoms than NFO by up-regulating the generation of Tregs. It is not yet clear why FFO has stronger immunomodulatory effects than NFO. So we are currently trying to identify the interrelationships between FFO, EPA, DHA and Tregs. Our results suggest that the anti-allergic effect of FFO is associated with the enrichment of CD4^+^CD25^+^Foxp3^+^ Treg at the site of inflammation and that fermented fish oil may be an effective treatment for the allergic symptoms of AD.

## Conclusions

In conclusion, we produced fermented fish oil and tested its ability to suppress the allergic inflammatory response and to activate CD4^+^CD25^+^Foxp3^+^ T cells. Administration of FFO alleviated the allergic inflammation, and did increase the expression of the immune-suppressive cytokines TGF-β and IL-10. Increased TGF-β and Foxp3 by FFO was associated with regulatory T cell populations, which can down-regulate the progression of experimental immune disorders. The ingestion of FFO up-regulated the generation of CD4^+^CD25^+^Foxp3^+^ Tregs. It is not yet clear why FFO has strong immune-modulatory effects. So, we are currently trying to identify the interrelationships between omega-3 fatty acids and Tregs. These results suggest that the anti-allergic effect of FFO is associated with enrichment of CD4^+^CD25^+^Foxp3^+^ T cells at the inflamed sites and that FFO may be effective in treating the allergic symptoms of AD.

## Methods

### Experimental animals

BALB/c mice (female, 7-weeks-old) were purchased from Orient Bio (Korea) and were maintained for 1 week before the start of any experiments. The mice were housed in the animal facility of Jeju National University under controlled temperature (23 ± 1°C), humidity (60 ± 10%), and light (lights on from 08:00 to 20:00 hours) and under pathogen-free conditions. All animal experiments were approved by the Animal Care and Use Committee at Jeju National University.

### Fermentation of fish oil

FFO was provided by Fermentec Inc. (Jeju, Korea). Pulverized fish by-product was mixed with water, raw sugar, *Lactobacillus plantarum* and *Saccharomyces cerevisiae* under anaerobic fermentation conditions. After 15 days, hexane was added to the fermented fish liquid. FFO was separated from the hexane extract by rotary evaporation.

### Fatty acid composition of natural fish oil and fermented fish oil

The fatty acid compositions of NFO and FFO were analyzed by the Feeds & Foods Nutrition Research Center (Pukyong National University, Korea) using the gas chromatography (Table 1).

### Determination of optimal dosage of FFO

To determine the optimal dose of FFO, we tested the anti-allergic effect of FFO in the compound 48/80 (Sigma-Aldrich)-induced pruritus mouse model. FFO (20, 50 or 100 mg/kg) was orally administered 1 h before the subcutaneous injection of compound 48/80 (5 mg/kg). One hour after injection of compound 48/80, the amounts of IgE and histamine in the serum were measured. A dose of 100 mg/kg of FFO reduced serum IgE and histamine levels in this model.

### DNCB application to induce experimental AD

Mice were divided into five groups (normal, induction, positive control, NFO and FFO; n = 8 mice per group). Mice were painted with 300 μL of 1% DNCB or vehicle on their abdomen as the first sensitization (day-7). On day 0, the mice were sensitized with 200 μL of 0.5% DNCB on their ears and were then sensitized with 200 μL of 0.5% DNCB every other day for up to 31 days. From day 12 until the completion of the experiment, mice were given FFO or NFO (100 mg/kg) in their drinking water, daily, and were painted with hydrocort cream (Green Cross, Korea) containing 2 mg/g hydrocortisone valerate as positive control on their ears every other day. The mice were sacrificed on day 32.

### Scratching behavior, macroscopic edema and histological evaluation

In the experimental AD mouse model, DNCB stimulation elicited scratching of the ears with the hind paws around the painted site. The mice in each group were placed in an observation chamber and scratching behaviors were videotaped for 10 min. The number of scratching behaviors during this period was counted. Ear thickness was measured using a Digital Thickness Gauge (Mitutoyo). Ear tissues were fixed with 10% formalin and embedded in paraffin. Paraffin sections (3 μm each) were stained with H&E.

### Immunohistochemistry (IHC) assay for detection of CD3^+^ cells and TSLP in ear tissue

To analyze the expression of TSLP in each ear, paraffin sections (3 μm, each) of ear tissue were prepared. IHC was performed with rabbit anti-CD3 and rabbit anti-TSLP (Novus Biologicals) and a rabbit-specific HRP/DAB detection IHC kit (Abcam) according to the manufacturer’s instructions.

### Western blot for detection of TSLP in ear tissue

Total protein was isolated from ear tissue using lysis buffer and the protein concentration of each sample was quantified by the bradford assay. The proteins were electroblotted onto a polyvinylidene difluoride (PVDF) membrane using an iBlot gel transfer device (Invitrogen). The membrane was incubation with rabbit anti-TSLP (Novus Biologicals). After washing, the membrane was incubated with horseradish peroxidase (HRP)-conjugated anti-rabbit IgG. The blot was visualized with a western blot detection system (iNtRON Biotechnology) according to the manufacturer’s instructions.

### Elisa

IgE (Biolegend, San Diego, CA) and histamine (Labor Diagnostika Nord, Nordhorn, Germany) in mouse serum and IL-4 and IFN-γ (R&D Systems, St. Louis, MO) in the supernatant of cultured cells were measured using ELISA kits according to the manufacturer’s instructions.

### Splenocyte culture in BALB/c mouse

Mice from each group were sacrificed by cervical dislocation and their spleens were removed aseptically. To obtain single-cell suspensions, red blood cells were removed using red blood cell lysis buffer. After washing, splenocytes (1.0 × 10^6^ cells/mL) were incubated in the presence or absence of anti-CD3 (1 μg/mL) and anti-CD28 (0.5 μg/mL) (eBioscience) for 3 days. Following incubation, the supernatants were collected to determine levels of IL-4 and IFN-γ and the cells were immediately analyzed by flow cytometric analysis (BD Biosciences).

### Isolation of CD4^+^ T cells from splenocytes

Isolation of CD4^+^ T cells from splenocytes used a purified anti-mouse CD4 (eBioscience) that had been labeled using the DSB-X^TM^ Biotin Protein Labeling Kit (Invitrogen) according to the manufacturer’s instructions. Briefly, cells were incubated with DSB-X labeled antibody for 10 min. The bead-bound cells were incubated for 10 min in FlowComp release buffer. The supernatant containing the bead-free cells was transferred to a new tube and the cell pellet was resuspended in RPMI Medium 1640 (Gibco).

### Flow cytometric analysis (FACS)

To analyze Foxp3 expression, cells were permeabilized with the Foxp3 fixation/permeabilization kit (BD Biosciences) and stained with anti-Foxp3-FITC (eBioscience) according to the manufacturer’s instructions. Briefly, to block mouse Fc receptors, the cell suspension was incubated with CD16/CD32 (BD Biosciences) for 15 min, followed by incubation in fixation/permeabilization buffer for 20 min and anti-Foxp3-FITC for 30 min. To measure the differentiation of CD4^+^CD25^+^ T cells, CD4^+^ T cells were stained with anti-CD4-FITC (eBioscience) and anti-CD25-PE (eBioscience). To block mouse Fc receptors, the cell suspension was incubated with CD16/CD32 (BD Biosciences) for 15 min, followed by incubation with anti-CD4-FITC for 30 min and anti-CD25-PE for 30 min.

### Extraction of total RNA and real-time PCR

Total RNA was isolated using TRI reagent (Molecular Research Center) according to the manufacturer’s instructions. Reverse transcription was performed using a First-Strand cDNA Synthesis kit (Promega). The real-time quantitative PCR was performed with TaqMan®Universial Master Mix II or Brilliant III Ultra-Fast SYBR® Green QPCR Master Mix (Agilent Technologies) with a StepOnePlus^TM^ Real**-**Time PCR (Applied Biosystems). Real-time PCR results were analyzed using the StepOne^TM^ software, which measures the amplification of the target and the endogenous control in samples and in a reference sample. The data were normalized using the endogenous control. The mRNA levels were normalized to the expression of GAPDH. The following primers were used: mIL-4 (forward: 5’-ACAGGAGAAGGGACGCCAT-3’; reverse: 5’-GAAGAACTACAGACGAGCTCA-3’), mIL-13 (forward: 5’-GCAACATCACACAGGACCAGA-3’; reverse: 5’-GTCAGGGAATCCAGGG CTAC-3’), mIFN-γ (forward: 5’-TCAAGTGGCATAGATGTGGAAGAA-3’; reverse: 5’-TGGC TCTGCAGGATTTTCATG-3’), mTGF-β (forward: 5’-GAAGGCAGAGTTCAGGGTCTT-3’; reverse: 5’-GGTTCCTGTCTTTGTGGTGAA-3’), mIL-10 (forward: 5’-ATAACTGCACCCAC TTCCCA-3’; reverse: TCATTTCCGATAAGGCTTGG-3’), and mFoxp3 (forward: 5’-CCCATCCC CAGGAGTCTTG-3’; reverse: 5’-CCATGACTAGGGGCACTGTA-3’)

### Statistical analysis

Quantity One version 4.2.1 and Image-Pro plus version 4.5 software were used to transform images into numerical values. Student’s *t*-test and two-way analysis of variance were used to determine the statistical significance of differences between experimental and induction groups. Data are shown as mean ± standard deviation.

## Abbreviations

AD: atopic dermatitis; NFO: natural fish oil; FFO: fermented fish oil; EPA: eicosapentaenoic acid; DHA: docosahexaenoic acid; TSLP: thymic stromal lymphopoietin; T-bet: T-box-expressed-in-T-cell; GATA3: GATA-binding protein 3; Foxp3: forkhead box P3; Tregs: regulatory T cells.

## Competing interests

The authors declare that they have no competing interests.

## Authors’ contributions

S-CH and G-JK designed methods and carried out most experiments and wrote the original manuscript; Y-JK performed ELISA, real-time PCR and the flow cytometric analysis; S-WM and Y-SA produced FFO and NFO; H-KK and E-SY supervised the studies and wrote the manuscript. All authors read and approved the final manuscript.
